# Experiences of Pre-Implantation Genetic Diagnosis (PGD) in Sweden: a Three-Year Follow-Up of Men and Women

**DOI:** 10.1007/s10897-017-0078-7

**Published:** 2017-02-12

**Authors:** Stina Järvholm, Ann Thurin-Kjellberg, Malin Broberg

**Affiliations:** 1 Department of Obstetrics and Gynaecology, Sahlgrenska University Hospital, Blå stråket 6, 413 45 Gothenburg, Sweden; 2 Department of Psychology, University of Gothenburg, Box 500, 405 30 Gothenburg, Sweden

**Keywords:** PGD, Men and women, Psychological experience, Follow-up, Qualitative research

## Abstract

Men and women with a hereditary genetic disease are faced with different options when they wish to become parents. One is pre-implantation genetic diagnosis (PGD) which is a combination of in vitro fertilization (IVF) and genetic analysis of the embryo before implantation. The present study focused on men and women’s psychological experiences of PGD three years after applying for PGD. Nineteen women and seventeen men (i.e. seventeen couples and two women) participated. The interviews were analysed by thematic method. *It is better to have tried* was identified as a master theme, under which came three underlying sub-themes, which had the following headings: *Practical experience of PGD, Psychological experience of PGD* and *Goals of PGD.* The results show that men and women three years after PGD are still psychologically affected by their experience. The men and women raised concerns that their relationship had been affected, both positively and negatively, and feelings of anxiety and depression still remained. Healthcare services should recognize the heterogeneous nature of the group being studied and therefore the need for counselling can arise at different times and in relation to different areas, regardless of the outcome of the PGD.

## Introduction

Men and women who want children and who know they have an increased risk of transmitting a genetic disease form a heterogeneous group, but they also share the experience of being faced with difficult choices. Currently it is possible for individuals who are affected by a genetic disease to undergo pre-implantation genetic diagnosis (PGD), which is a combination of in vitro fertilization (IVF) and genetic analysis of the embryo before implantation. PGD makes it possible for couples to start a pregnancy with a reduced risk of transmitting a known genetic disease to the child (Liebaers et al. 2010). In Sweden, as in many other developed countries, approximately 4% of all children are born after IVF (Ferraretti et al. 2013). In a study of outcomes after PGD (also including pre-implantation genetic screening) including 1498 couples, it was found that 29% of the couples who had undergone a maximum of six PGD cycles, became parents after PGD treatment (Verpoest et al. 2009). The success rate differs, and the types of genetic conditions and the age of the woman at the time of PGD are the most significant predictors of success.

PGD is a relatively recent option and knowledge about the psychological aspects of the procedure is limited but increasing. A review by Hershberger and Pierce (2010) showed that the decision-making process for couples considering PGD includes cognitive appraisals in relation to risks, cost and time, emotional responses such as pain and joy, and moral judgments relating to social significance and disease prevention. Other studies have found that the decision to undergo PGD is a dynamic process occurring over time and involving a series of choices, where the individual moves back and forth in different dimensions that can be named *identify*, *contemplate*, *resolve* and *engage* (Hershberger et al. 2012). Both men and women are engaged in the decision to undergo PGD and their decision-making has been found to be related to themselves, existing or unborn children and their environment (Järvholm et al. 2014).

In a review of the psychological impact of PGD, Karatas et al. (2010) conclude that most studies have focused on women’s experiences and less is known about emotional reactions in men. In a recent study, men planning for PGD reported significantly more symptoms of anxiety than men planning for standard method IVF and the presence of an existing child with the genetic disease was a significant predictor of anxiety during the process (Järvholm et al. 2016). Among women, anxiety and depression seemed to fluctuate during the process but after successful PGD, levels returned to baseline at 24 weeks of gestation (Karatas et al. 2011). Many men and women applying for PGD are under strain posed by the genetic disease in themselves or their families, whether this takes the form of miscarriages, termination of pregnancies and/or having previous children with the disease (Bayat et al. 2011; Korenromp 2009; Lok and Neugebauer 2007).

### Aim and Research Questions

The aim of this study was to investigate long-term psychological experiences of PGD for men and women, including how their life situation and the PGD procedure affected their relationship and, three years after applying for PGD, how they coped with parenthood or childlessness.

## Material and Methods

### Participants

In Gothenburg, Sweden, in 2010 and 2011 men and women in the PGD group were recruited consecutively by their gynaecologist at their first visit to the reproductive medicine clinic. Participants had opted for PGD but had not yet started treatment. Twenty-two couples were eligible. Nineteen women and seventeen men agreed to participate (i.e. seventeen couples and two women). Reasons given for non-participation were “lack of time” and “it is too hard to think about the questions”. Ten of the couples carried monogenetic diseases, for example Dystrophia Myotonica, Fragile X, and severe metabolic diseases, and nine couples carried chromosomal disorders. Since pre-implantation genetic screening (PGS) is not accepted by law in Sweden no couples were currently applying for PGS. Seven of the couples lived in rural areas and twelve in urban areas. Four women and three men were first or second generation immigrants.

**Fig. 1:**
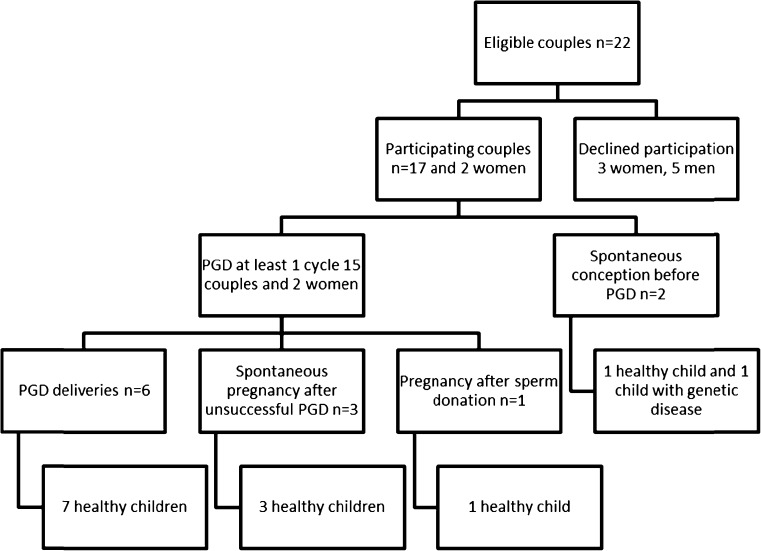
Pregnancy results three years after applying for PGD

After three years all participants had tried to achieve pregnancy, as seen in Fig. 1.

Fifteen of the couples and the two women had undergone at least one PGD cycle and six children (four singletons, one set of twins) had been born after PGD. For two couples it was their first child and for four of the couples it was their second or third child. The two couples who had not undergone PGD achieved spontaneous pregnancies before starting the PGD treatment, resulting in one healthy child and a child with the known genetic disease. In addition, three children were conceived spontaneously during the three years and in all cases involving monogenetic diseases, mothers underwent prenatal diagnosis and the children were born without the known genetic disease. One child was born after donation treatment. One couple were accepted for adoption but had not yet received their child after three years. Two women and one man had experienced the loss of a child due to genetic disease before applying for PGD; another couple lost their child before the three-year follow-up. Four couples were still undergoing PGD treatment after three years, three of the four making attempts to produce a sibling for a child born after a previously successful PGD. For one of the two women participating in the study it was a new round of PGD treatment with a new partner. This woman was the only one who had separated from her original partner during the three years. Participants’ characteristics are shown in Table 1.

**Table 1:** Participants’ characteristics at follow-up

Variable	Female (*n* = 19)	Male (*n* = 17)
Age		
Mean (SD)	34.3 (4.1)	33.8 (4.9)
Years in relationship		
Mean (SD)	12.58 (4.36)	12.88 (4.36)
Highest level of education		
Secondary school	4 (21.1%)	5 (29.4%)
University	15 (78.9%)	12 (70.6%)
Employed/self-employed	16 (84.2%)	17 (100.0%)
Parent when applying	6 (31.6%)	7 (41.1%)*
Parent three years after	12 (63.2%)	11 (64.7%)

*One man had a child from a previous relationship

### Procedure

The first author contacted the men and women by letter informing them that the three year follow-up interview was coming up. After this they were contacted by phone and asked if they were willing to participate. All nineteen women and seventeen men agreed to the follow-up interview. The first author interviewed the participants individually. About half of the participants came to the hospital for the interview and half preferred to be interviewed in their homes, mainly because of the need to take care of children or because they lived far from the hospital. The lengths of the interviews for women were 19 to 53 min (Mean 31 min) and for men 13 to 45 (Mean 30 min). The interview followed a semi-structured guide with four themes: (a) psychological aspects (b) experiences of the decision-making process regarding their wish for children, (c) experiences of PGD and (d) ethical and religious considerations. The interview was recorded and transcribed verbatim. The study was approved by the Ethics Review Board in in Gothenburg, Dnr: 300–09. Informed consent was obtained from all individual participants included in the study.

### Analyses

The material was analysed inductively using thematic analysis, in accordance with the methods of Braun and Clarke (2006). This method was chosen because the approach was explorative, with the aim of increasing knowledge about individual experiences of PGD. ATLAS.ti 7.5.7 was used to facilitate the qualitative data analysis.

All the authors familiarized themselves with the data by reading the interviews and noting their first impressions. Thereafter, the first author created initial codes and organized these into three broad themes: *1) the experience of PGD, 2) goals, 3) strains and gains,* with a total of 19 underlying categories. In the next stage of the analysis, the three themes were restructured into four main themes: *1) practical experience of PGD, 2) emotional experience of PGD, 3) psychological aspects and 4) goals*, which in total had seventeen sub-categories. After this, the first and third authors worked together on the model and the authors agreed on a final understanding of the themes and sub-categories shown in Table 2. *It is better to have tried* was identified as a master theme, affecting the three underlying sub-themes, which were labelled as follows: *Practical experience of PGD, Psychological experience of PGD and Goals of PGD* with 11 underlying categories. The quotations presented are identified by gender and a number indicating from which couple the participant comes (1–19).

**Table 2:** The data analysis; master theme, main themes and underlying categories

It is better to have tried, no matter what	
1. Practical experience of PGD	1.1 Opportunities
	1.2 Difficulties
	1.3 Relationship with healthcare service
2. Psychological experience of PGD	2.1 Development
	2.2 Deterioration
	2.3 Never give up
	2.4 Marital relationship
	2.5 Childlessness
	2.6 Ethical considerations
3. Goals of PGD	3.1 Parenthood
	3.2 To be like everyone else

## Results

The three sub-themes and their eleven underlying categories are presented in Table 2 below.

### It Is Better to Have Tried, No Matter What

Although all participants had decided on PGD, not all went through with it. Two couples achieved pregnancy spontaneously before starting PGD and only five (out of nineteen) women gave birth to a child after PGD. However all had been involved in PGD and felt they had gone through the PGD process. Before starting PGD both men and women were involved in questions that could be categorized as: practical (“What shall I do?”) philosophical (“What shall I think about it?”) and psychological (“How does it make me feel?”) (Järvholm et al. 2014). Three years after, they still struggled with the same questions although the first one had changed to “What have I done?” The answer to that question was “I have tried” and that answer affected the response to the underlying themes and was important in the participants’ psychological understanding of the experience of PGD, regardless of the actual process and outcome.

*“If there is the opportunity, there is no reason not to go through with it… I can hardly see that you would regret it… But I reckon that that’s sort of my attitude - that you seldom regret something that you do, more likely the things that you don’t do.” Male (couple 19)*

### Practical Experience of PGD

Both men and women addressed the practical pros and cons associated with PGD, such as the opportunity to avoid terminations, or difficulties planning everyday life during treatment and the imposition of medication. They were also able to address the fact that the practical aspects of PGD are a mix of opportunities and difficulties, and this causes ambivalence.

*“ PGD felt like a fantastic way out of everything… and yet it was a much tougher process than I could imagine and that I was informed about…” Female (couple 19)*

#### Opportunities

Both men and women described the opportunities offered by PGD in terms of having a healthy child, or at least trying their best to change life back to the “original plan,” as if the PGD attempt was the road back to life as it was meant to be.

*“…It really felt like now we were being offered the best there was and we really tried to make it work… and if you do that, then it should work. Nothing could go wrong if you pick (the best embryo) and try hard. It… felt in that way that it was a proper attempt.” Male (couple 13)*

Both the participants with experience of terminations and those without such experiences expressed the opinion that PGD was a possible solution to avoiding termination (later on) in pregnancy and this was a central aspect of choosing PGD.

*“ Well, it (termination) was something that we would like to avoid. Because it just feels much harder to be pregnant for a long time and then terminate the pregnancy and it takes much longer. Therefore we thought that PGD was a better option.” Male (couple 17)*

The focus on opportunities offered by PGD may lead to unpreparedness for the practical and emotional stressors associated with the treatment. The men and women participating expressed the feeling that PGD was an external solution which was provided for them and was controlled by outside agencies.

#### Difficulties

The participants described the negative practical aspects of the treatment process, such as procedures regarding medication, negative consequences on everyday life and the results of outcome failure.

*“The hormonal treatment is no fun, so to speak. And that you were supposed to do it while you were working. … and then we live in another town… and trips to the hospital were always badly timed when you were planning something else … so in that way it is hard and still is – but this is something that we had chosen.” Female (couple 18)*

The participants also described the stress associated with practical aspects of the treatment.

*“…powerlessness, not being able to do anything but take the injections and so. It was difficult. Regardless of whether you took good care of your body and were doing your best it was not possible to get a good result (embryo). You couldn’t do anything but take the drugs. That was hard. To get the information. The second time it worked better but we got an embryo that didn’t take. Well… it didn’t work. And yet we had such high expectations…” Female (couple 12)*

#### Relationship with Healthcare Service

The men and women in the study had several contacts with healthcare services regarding their wish for children. They described positive experiences such as good treatment, feelings of being “special” and working together as a team.

*” It felt nice that finally someone took you seriously… with the miscarriages it was messy and hard to get help from the healthcare services, so to say… but then (at the PGD unit) it felt good to get started and to get help.” Male (couple 18)*

Negative experiences were often associated with lack of information before couples reached the specialized PGD unit, but also during the treatment at the unit. Lack of information also made planning and decision-making more difficult. Both men and women showed ambivalence when talking negatively about healthcare services. After expressing criticisms it was common for them to add that they understood the position of healthcare staff.

*” The information is not enough and it seems that important information is lacking … They are professionals. They know what they are doing. But still information is lacking, regarding our age, especially hers and that kind of thing, it should have gone faster. But still I am grateful, they tried.” Male (couple 10)*

### Psychological Experience of PGD

The experience of PGD was associated with a mix of emotions, and both positive and negative psychological experiences. A common perspective three years after initiating PGD was that people have to cope with the fact that they have “chosen” PGD and “have to” keep on doing it even when it is experienced as painful.

*“It is a rollercoaster. Sometimes I feel awful and sometimes I feel pretty good, but the rollercoaster has been less extreme lately. There are not so many peaks and the worst valleys are gone, but it is more, like … it has levelled out. In both good and bad ways. This protects you a bit but of course you would like to keep the peaks” Female (couple 13)*

#### Development

Both men and women reported positive psychological experiences during the process of PGD. They talked about increased patience, open mindedness and the ability to relax and cope with difficulties.

*“I feel very well. I feel… I am often happy, I am really positive, but I can also feel very low some times, but it doesn’t last very long and I am not afraid of feeling down. I think this has been the best experience that I could take with me from this process (PGD) … that also when I feel really, really down I am still able to find some positive things after all.” Female (couple 9)*

#### Deterioration

Both men and women said that their wish for a child in general, and the period of treatment, had affected them negatively. A negative psychological state, deterioration, was related to not being able to control the situation and facing an unknown future.

*“Then I experienced when I sat there … that you are totally helpless. If something doesn’t work properly I am used to being able to put it in order. Then I have control. …My partner was in pain and I felt absolutely powerless. I couldn’t do anything, I didn’t know what was happening, I was completely excluded … I experienced such stress all of a sudden, I felt like I was going to be sick.” Male (couple 6)*

Also the experience of feeling depressed was common.

*“It has not been fun. I have been affected as a person. I am not at all the same person that I was before this started… I am not at all, how should I say? Happy is maybe wrong, but I am not at all… I don’t get happy or cheerful as easily (anymore).” Female (couple 18)*

Neither men nor women were spared from other everyday stressors that occurred simultaneously with the PGD process.

*“…I feel really down, actually, but it is not just this, there are also other things. It is hard at work and there are other disasters, it kind of puts extra burdens on me. It kind of makes it even more difficult to cope and I tend to try not to focus on the family situation right now. It is hard to say if it this or that makes you miserable, but it is for sure heavy. Really heavy.” Male (couple 13)*

#### Never Give Up

This theme was a mix of hope and realism. On one hand it was important not to lose faith that PGD was going to work. This approach often resulted in partners continuing the PGD treatment just one more time.

*“It has been hopeful every time, you think “this time it will be a child”, but it doesn’t happen.” Female (couple 17)*

On the other hand there was also a feeling that this strategy resulted in other options being put on hold for too long, and that the time perspective in the process got out of hand.

*“Now when you see that everything takes such a long time you should have started everything earlier. More parallel tracks, more often and sooner.” Male (couple 13)*

#### Marital Relationship

The participants described how the period of struggling with different ways to achieve parenthood had affected their marital relationship. There were descriptions of how the relationship was strengthened, but also put under strain.

*“I think that we have become closer, we have been through things that most people haven’t. We have got to know each other, and what shall I say, different sides than most people get to know about their partner, I think. So in many ways we have become closer.” Female (couple 13)*

There were also concerns raised about how the partner was affected by the situation. These concerns were more often expressed by the men and associated with the IVF treatment and its impact on the woman. This concern was more strongly expressed when the man carried the disease.

*“This was almost the toughest thing during the whole process knowing, that it is me that is causing this - it is not me causing it in that way, but sometimes I think that X has to go through with all this, just because she has chosen to live with me, and that feels … or that has been feeling very tough sometimes, that she has to put up with all this.” Male (couple 18)*

Both men and women described the man as being in a position outside the main events. This could be seen both on an individual level and as a possible relational strain.

*“She was in control of most things…I think she had more control of circumstances, looking back I think I maybe could have been a better support. I don’t know how but … I didn’t think of it then. I was too caught up in myself, maybe…” Male (couple 12)*

#### Childlessness

Both the couples who were childless and those who were in treatment for a sibling expressed the opinion that other couple’s pregnancies were hard to handle. This was more strongly expressed among the participating women and affected everyday life in terms of avoidance of social situations reminding them of their childlessness.

*“This has almost become an obsession at times; it is such a strong wish, almost like you were childless and just longing for a child… I am really happy that we have C, that we got her, and if it is just her, if it is just going to be her I have slowly to come to accept this, but it is almost like it is stuck in my head that she has to have siblings and that we need one more, only then we will be complete.” Female (couple 1)*

#### Ethical Considerations

The participants considered the ethical dimensions surrounding PGD from several perspectives. One was the understanding that others could think of PGD as strange and unnatural and that the use of PGD could mean that society was on an ethical slippery slope. Also the cost of publicly-funded treatment was taken into account. A common position was to state that PGD is ethical since it does not focus on traits but is only to be used to avoid disease.

*“… others can think that you are customizing a child. I find that position a bit hard to understand, because it is a disease you are excluding through PGD. It is not like you are trying to create an elite athlete or something…” Male (couple 14)*

Participants also had the view that if they had not tried to stop the occurrence of the known disease and instead given birth to a child with a severe disease, this would have been more unethical than the use of prenatal testing.

*“…You can never judge another person’s decision and it has to be…right for that individual. I don’t think you can judge and I am totally confident that we have made the right decision for us. Others can think differently and make other choices and then it is right for them. But seeing how my child felt and how difficult it was for him I could never again give birth to a child with that disorder knowing how it is, when I have the opportunity to check this in advance” Female (couple 9)*

### Goals of PGD

The goal of PGD - to put a stop to a known disease - was described in the same way regardless of whether the couple had achieved parenthood or not through PGD. The men and women who were childless after three years still expressed loyalty to the goal.

*“To get a chance to have a child without this gene, and with proof that the child is not likely to have this gene. That felt really good.” Female (couple 19)*

The goal of PGD was also related to the future and the hope that the child as an adult could be spared the strain of decision-making associated with prenatal testing.

*“It was the best decision we have ever made, I think. Now we know. Our child is healthy… and if he wants to have children he doesn’t have to think about this. We have put a stop to the disease…” Male (couple 8)*

#### Parenthood

For all the men and women engaging in PGD the obvious goal was a (healthy) child. Twelve of the couples had at least one child during the three years after applying for PGD but only for five of them was the child a result of PGD. Regardless of whether PGD treatment resulted in a birth the decision to undergo PGD was described as a part of the road to parenthood. And the child born was described as an extra special child.

*“It took a year before I could believe that it was true, that he existed. Can it be this good? I could just look (at him) and be totally astonished and think; ‘We must take extra care of him because he is not re-makeable…” Female (couple 2)*

For those who achieved parenthood for the first or second time, this was also strongly connected to the feeling of being like everyone else.

#### To Be like Everyone else

To move from being “different” to being like everyone else was described as joyful and a relief. But the transition was also associated with mixed emotions.

*“…and it was a very peculiar situation that suddenly you didn’t have anything sad to worry about… but that was not only positive. Because you no longer have ups and downs…I don’t know… but I think that sometimes if you have something sad in your life you can more strongly appreciate things that are positive …” Female (couple 2)*

And for those not achieving parenthood, they compared themselves with the imagined version of themselves and how life should have been, in comparison to others.

*“It was like you had a different picture of the future, I mean how it should have been perhaps … if you had been “like everyone else” you should have had a child or two right now…” Female (couple 5)*

## Discussion

The aim of this study was to investigate the long-term experiences of PGD in men and women. The group was heterogeneous when applying for PGD and even more heterogeneous in their experiences after three years. However, when applying for treatment, all felt that they had just undergone a decision-making process with emotional, cognitive and practical dimensions (Hershberger et al. 2012; Hershberger and Pierce 2010; Järvholm et al. 2014). Three years after applying, all men and women in the present study reported that they had tried to achieve pregnancy and seventeen out of nineteen women had undergone at least one PGD cycle. Four were still in contact with healthcare services regarding their wish for a child and only five had given birth after PGD. The broad diversity of experiences among these men and women is interesting in its full complexity, and cannot be reduced to generalized statements including such terms as “always” or “never”. It is difficult to say what changes are caused by PGD and what are caused by other circumstances. Nevertheless the strength of the present study is that it conceptualizes common themes despite the different experiences of the group, and gives an in-depth understanding of their situations.

The master theme *It is better to have tried, no matter what,* can be seen as an active, problem-focused, coping strategy. Previous studies have shown that active coping is associated with strength and the ability of men and women to handle the stress of IVF treatment (Gourounti et al. 2012; Peterson et al. 2006). PGD is a combination of IVF and prenatal diagnosis and therefore more complex than IVF only. The advantages associated with an active coping strategy are consistent with previous work about coping and IVF treatment; those with active coping strategies better manage the situation. An active coping strategy could be a trait that is needed for people to find, and go through with, PGD. It is also allied with the sub-theme *Never give up* where participants described the negative side of this strategy, when it put other options on hold, prolonged the process of adjustment to childlessness, and put the focus on the demanding process to shift between different coping strategies.

Men and women seem to be affected differently by *Childlessness,* and their perceptions of the *Marital relationship* also seem to differ. Women in the study described more distress from being childless than the men did. This is consistent with previous work (Huppelschoten et al. 2013; Preedy and Watson 2010). Regardless of whether the women participating were childless or in treatment for a sibling they both experienced strain when exposed to other couple’s pregnancies. Feelings of tension when faced with other couple’s pregnancies are not only linked to childlessness but could be related to marital satisfaction or feelings of depression. In the *Marital relationship* sub-theme both men and women stated that they had been affected by PGD in different ways. The women described more stress relating to the IVF procedure, and the men more stress relating to feelings of exclusion. It is well-known that stress levels in women facing IVF treatment are elevated, while men tend to be less affected (El Kissi et al. 2013; Wichman et al. 2011). For men and women undergoing PGD the situation resembles ordinary IVF treatment but also differs from it. The PGD couples could be more equal affected by the burden of disease and/or a more troubled reproductive history, which could contribute to the common theme found in both men and women in the PGD group, and is consistent with previous studies (Järvholm et al. 2016). The choice of PGD is made jointly since it is a prenatal option before pregnancy (Zeiler 2007).

The goal of PGD is of course to become a parent of a child without the genetic disease. Before the start of the PGD treatment it seems like the perfect solution. But it is a demanding treatment in combination with a demanding life situation before treatment, and therefore PGD is probably most well-suited for self-selected couples with psychological strengths. The men and women in the present study described both practical and emotional strain continuing for years, and the experience of PGD kept on affecting them in both positive and negative ways. It became clear that the goal was not just to have a child; it was also to put a stop to the disease and to prevent the child from being in the same situation in the future. Having a healthy child was also a way the parents could become like everyone else.

### Study Limitations

The study has several strengths; to the best of our knowledge this is one of the longest follow-ups of patients undergoing PGD, with no drop-outs. For a qualitative study with in-depth interviews it consists of a large number of participants, both men and women. However the study is limited by including participants from only one clinic. Furthermore, the results could have been affected by the research being conducted by the psychologist at the clinic, possibly limiting the participants’ raising of negative perspectives regarding healthcare services they had experienced there. However, we make no general claims; the results of the present study add diversity and in-depth understanding of the experiences of the men and women interviewed and it is fair to believe that their experiences are shared by other individuals undergoing PGD. However, there could also be other experiences that have not been captured in the present study.

### Practice Implications

The results show that men and women three years after PGD are still psychologically affected by their experience. It is reassuring to see that many individuals became parents through PGD or by other options, that everyday life for many seems to function as it did before PGD, and that couples stayed together. However, stability is not the same as psychological well-being. The men and women in the study raise concerns that their relationship has been affected, both positively and negatively, and feelings of anxiety and depression still remain. Others are still involuntarily childless. Healthcare services should recognize the heterogeneities in the group and the fact that treatment may continue for several years. Therefore the need for counselling can occur at different times and may apply to different areas, regardless of the outcome of the PGD.

### Research Recommendations

Three years were not enough to capture all participants’ experiences after PGD, some were still in treatment at that point. Therefore a longer time to follow-up would have increased knowledge even more. Then it would also be possible to gained knowledge about the 1/3 that who at year three still not were parents and learn more about their mental well-being and choices. Studies using validated scales to measure psychological impact could be used in further studies to improve understanding of this group.
